# Characterisation of the Molecular Mechanism of Permeation of the Prodrug Me-5ALA across the Human *Stratum Corneum* Using Molecular Dynamics Simulations

**DOI:** 10.3390/ijms232416001

**Published:** 2022-12-15

**Authors:** Janonna Kadyrov, Lanie Ruiz-Perez, Heather A. E. Benson, Ricardo L. Mancera

**Affiliations:** 1 Curtin Medical School, Curtin Health Innovation Research Institute and Curtin Institute for Computation, Curtin University, GPO Box U1987, Perth, WA 5845, Australia; 2 Basil Hetzel Institute for Translational Health Research, 37a Woodville Road, Woodville South, SA 5011, Australia; 3 UniSA Clinical and Health Sciences, University of South Australia, Adelaide, SA 5001, Australia

**Keywords:** transdermal drug delivery, drug lipidation, prodrug, drug permeation, lipid bilayer

## Abstract

The barrier imposed by the outer layer of the skin, the *stratum corneum*, creates an almost impermeable environment for exogenous substances. Few lipophilic drugs with low molecular mass can passively diffuse through this layer, highlighting the need to develop methods to enable the delivery of more drugs via the transdermal route. The prodrug approach involves modifying the structure of a drug molecule to enhance its permeability across the skin, but it is often difficult to predict how exactly changes in chemical structure affect permeation. This study uses molecular dynamics simulations to predict permeability values and adequately characterise the molecular mechanism of permeation of the prodrugs Me-5ALA and its parent compound 5ALA across a molecular model of the lipid bilayers of the human *stratum corneum*. The influence of increased hydrophobicity in Me-5ALA on its permeation revealed a reduction in hydrogen bonding capability that enables it to interact more favourably with the hydrophobic region of the bilayer and diffuse at a faster rate with less resistance, thus making it a better permeant compared to its more hydrophilic parent compound. This molecular simulation approach offers a promising route for the rational design of drug molecules that can permeate effectively across the *stratum corneum*.

## 1. Introduction

Dermal and transdermal drug delivery (TDD) involves the administration of a drug to the surface of the skin, which is an easily accessible option that offers many advantages compared to other commonly used routes [1,2]. However, dermal/transdermal drug delivery is challenged by the fundamental structural and functional properties of the skin, which acts as the primary barrier against permeation of external substances and tightly regulates the loss of water [1,3].

The barrier property of the skin is attributed to the structural characteristics of the outer layer, the *stratum corneum* (SC), which is conceptualised as having a “bricks and mortar” structure [4]. The bricks represent the dead, keratin-filled corneocyte cells, and the mortar represents the surrounding lipid matrix, which is organised into stacked lipid bilayers [1,4]. This lipid matrix has a highly complex lipid composition, but is primarily comprised of ceramides (CER), cholesterol (CHOL) and free fatty acids (FFA) [5]. The primary pathway for molecules to translocate across the SC is by passively diffusing through the lipid matrix; however, such molecules must be lipophilic with low molecular mass (<500 Da) and low dosage requirements (<10 mg per day) [1,2,3]. Thus, the main challenge faced in TDD is the development of drugs that are able to permeate through the SC and reach their site of action, whilst preserving the integrity of the active drug component [6].

One method to enhance transdermal permeation of drug molecules involves optimizing the structural and physicochemical properties of the drug or formula, such as the use of chemical enhancers, transport vehicles and prodrugs [7,8,9]. The prodrug approach involves altering the structure of the drug to optimise its physicochemical properties for skin permeation, rather than addition of a chemical agent that acts by compromising the structure of the skin [10,11]. Examples of this are methyl-5-aminolevulinate (Me-5ALA), the methyl ester derivative of 5-aminolevulinic acid (5ALA) [12,13]. The prodrugs Me-5ALA (Metvix^®^) and its parent compound 5ALA are used in photodynamic therapy for the treatment of non-melanoma skin cancers, in particular actinic keratosis and basal cell carcinomas, the management of inflammatory acne vulgaris and sebaceous gland hyperplasia, and for photo rejuvenation of the skin [12,13]. They are applied topically in a cream or gel and when sufficiently absorbed through the SC to reach the target sites in the viable tissue, are metabolised into the photosensitiser agent protoporphyrin IX (PpIX) [14]. The application of red light then activates the PpIX molecules to interact with molecular oxygen to produce cytotoxic singlet oxygen [14]. As red light is applied, PpIX accumulates cytotoxic oxygen species in the skin lesions, which causes cell death [12,14]. The hydrophilic molecule 5ALA, has poor passive permeability, and therefore requires a high dosage and long administration times. Addition of an extra methyl group to form Me-5ALA [15,16] increases the lipophilicity of the methyl ester prodrug, thereby facilitating permeability through the lipid matrix of the SC, as well as shortening the application period prior to light activation [12,13,16].

The development of prodrugs is a complex process that faces a number of challenges, including inconsistency in measured permeability coefficients [6,15]. This is largely due to the highly complex lipid composition found in mammalian SC, which varies between and within individuals, and also the variability in in vitro permeation testing (IVPT) protocols used to determine skin permeation that make it difficult to compare permeability coefficients [17,18,19,20,21]. For example, the permeation of 5ALA has been studied using human skin from different anatomical sites, animal skin models, a range of diffusion cell types, receptor solutions and preparation protocols. Studies utilising human skin from the thigh and abdominal regions, in both horizontal and vertical diffusion cells, reported 5ALA to have skin permeability of 10^−13^ nm/ps [22,23]. The permeation of Me-ALA was later studied using a Franz-type diffusion cell on a piglet skin sample and reported to have a transdermal permeability of 10^−13^ nm/ps in both solution and gel [24]. Enhanced skin permeation using microneedles [25,26], electrical [27,28] and ultrasound energy [29] and novel formulations [30,31] has also been reported. However, many of the studies of 5ALA and Me-5ALA focus on PpIX levels achieved and clinical outcomes rather than direct measurements of skin permeation. The permeation of both 5ALA and Me-5ALA has also been studied using molecular dynamics (MD) simulations using a pure phospholipid (dipalmitoylphosphatidylcholine, DPPC) bilayer, and both drug molecules were reported to have a transdermal permeability of ~10^−4^ nm/ps [15]. This large discrepancy between experimental and simulation determinations highlights the importance of using a more realistic molecular model of the lipid layers of the SC.

MD simulations of drug transport through the SC lipid bilayers are directly focused on characterising the molecular mechanism of permeation of drugs, accounting for local forces, membrane structure and rate of diffusion of the drug across SC lipids [20]. This reflects the fact that permeation of drug molecules depends on their SC partition coefficient and diffusivity, which determine the SC permeability coefficient [1]. One advantage of MD simulations is that they can directly model changes in drug diffusion caused by variations in lipid composition, temperature, pH and hydration levels [32], as well as the presence of penetration enhancer chemicals and vehicle components [20]. This is possible because the prediction of structural, thermodynamic and dynamic properties of individual molecules as well as ensemble averages allow MD simulations to provide exquisite atomistic detail that experimental methods are unable to do [33]. Furthermore, characterisation of changes in free energy and the prediction of partition, diffusion and permeation coefficients, as well as changes in molecular structure and interactions, enable elucidation of the mechanism of permeation of drug molecules across lipid bilayers [18,33,34,35].

Understanding the physicochemical properties of the skin, in particular the SC, and its interactions with drugs and other small molecules is of great relevance in the fields of pharmaceutical and cosmetic product development and aids in the understanding and care of healthy and diseased skin [9,11,36]. It is thus highly desirable to determine if MD simulation methods can accurately predict the permeability of drugs through validation with experimental data, thus providing a more convenient approach for the development and testing of drugs for dermal and transdermal use. This work describes the successful use of MD simulation approaches to characterise the permeation of 5ALA and its methyl ester derivative (Me-5ALA) across a molecular model of the lipid bilayers of the SC to rationalise the influence of chemical structure on the mechanism of permeation. The permeation of Me-5ALA is indeed predicted to exhibit better permeation than 5ALA due to a reduction in its hydrogen-bonding interaction potential.

## 2. Results

**Figure 1:**
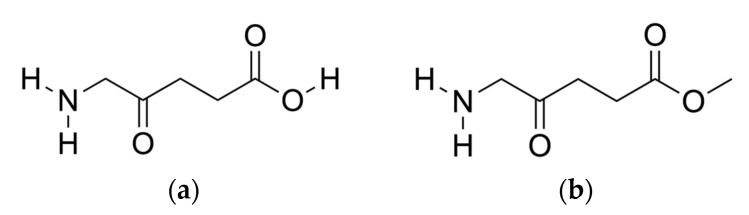
Chemical structures of: (**a**) 5ALA; (**b**) Me-5ALA.

Figure 1 shows the chemical structures of 5ALA and Me-5ALA. The additional methyl group in the prodrug Me-5ALA compared to its parent compound 5ALA, increases the molecular weight and lipophilicity as well as adds one rotatable bond, but also reduces the number of H-bond donors from two to one. This reduces the ability of Me-5ALA to form H-bonds with other molecules [15]. We interpret our subsequent findings in light of these simple differences between these two drug molecules.

### 2.1. Predicted Free Energy Profiles

**Figure A1:**
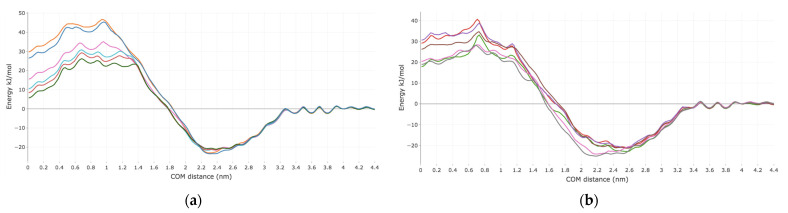
Convergence of the predicted PMF: (**a**) for Me-5ALA the 50–100 ns trajectory is shown in orange, 100–150 ns in dark blue, 150–200 ns in pink, 180–230 ns in light blue, 190–240 ns in red and 200–250 ns in green; (**b**) for 5ALA the 50–100 ns trajectory is shown in purple, 100–150 ns in red, 150–200 ns in brown, 180–230 ns in green, 190–240 ns in pink and 200–250 ns in grey.

The computed potential of mean force (PMF) of Me-5ALA converged after 210 ns of simulation time, whilst the PMF of 5ALA did so after 190 ns. The progressive changes in the computed PMF until convergence is reached for each permeant are reported in Figure A1 in Appendix A. The time that each simulation took to converge was discarded for further analyses, such that the production time for the US+REST3 simulations was 210–250 ns (i.e., the final 40 ns) for Me-5ALA and 190–250 ns (i.e., the final 60 ns) for 5ALA. These production periods were used for all subsequent calculations.

**Figure 2:**
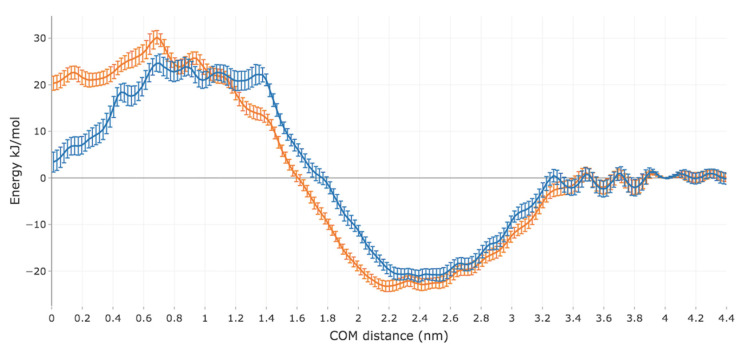
Potential of mean force (PMF) of the permeation of Me-5ALA (blue) and 5ALA (orange) through a model of the lipid bilayer of the SC.

The converged PMFs for the permeation of Me-5ALA and 5ALA are shown in Figure 2. It can be seen that as Me-5ALA approaches the bilayer surface, its PMF decreases and reaches a minimum value of −21.1 kJ/mol within the hydrophilic lipid headgroup region at a distance of 2.4 nm from the centre-of-mass (COM) of the bilayer. The PMF then exhibits an energy barrier, with the free energy increasing as the drug permeates through the high-density hydrophobic lipid tail region, reaching a maximum value of 24.5 kJ/mol at a distance of 0.7 nm from the COM of the bilayer. This is followed by a substantial decrease in the PMF to a value of 3.4 kJ/mol when Me-5ALA reaches the middle of the bilayer (at a COM distance of 0 nm). A qualitatively similar trend is observed for the permeation of 5ALA through the bilayer, but with some important differences. 5ALA exhibits a somewhat lower free energy minimum of −23.0 kJ/mol at a closer distance of 2.2 nm from the COM of the bilayer, followed by a higher free energy barrier with a maximum of 30.2 kJ/mol at a COM distance of 0.7 nm. The PMF then decreases as 5ALA approaches the bilayer centre to a value of 20.3 kJ/mol at the COM.

These predicted free energy profiles reveal that the interactions between both the drug molecules and the bilayer are most favourable in the hydrophilic lipid head region, as expected, and then become progressively unfavourable as the drug molecules travel through the high-density hydrophobic tail region, where they experience the most energetically unfavourable interactions with the bilayer. The more substantial drop after the maximum free energy barrier indicates that the interactions between Me-5ALA and the lipid bilayer in the low density hydrophobic region are favoured, whereas the interactions that 5ALA has with the bilayer in this region remain highly unfavourable because the free energy barrier remains high. This suggests that 5ALA interacts with the bilayer unfavourably during the entire translocation through the hydrophobic lipid tail region, and there is a significant difference in the molecular interactions between 5ALA in the bilayer compared to Me-5ALA that enables the latter to have a substantially lower relative free energy in the lower density interdigitating region. 

As indicated earlier, 5ALA has an additional H-bond donor, which makes this drug molecule more capable of H-bonding with the bilayer, which could explain the lower free energy minimum and better affinity for the hydrophilic head region compared to Me-5ALA, which has lost a H-bond donor as a result of the added methyl group. This could also be the reason why 5ALA has a much higher free energy barrier across the hydrophobic regions of the bilayer. The overall PMF trends observed for 5ALA and Me-5ALA agree qualitatively with previous simulations using a simple phospholipid bilayer, where favourable minimum and unfavourable maximum free energy values in the same regions observed in this study were reported, as well as that the interactions between the lipid tail region and Me-5ALA were more favourable than with 5ALA [15]. However, as the membrane model used in that work was a pure DPPC bilayer, the specific free energy values cannot be used to validate the predictions made in this study.

### 2.2. Diffusivity Profiles

**Figure 3:**
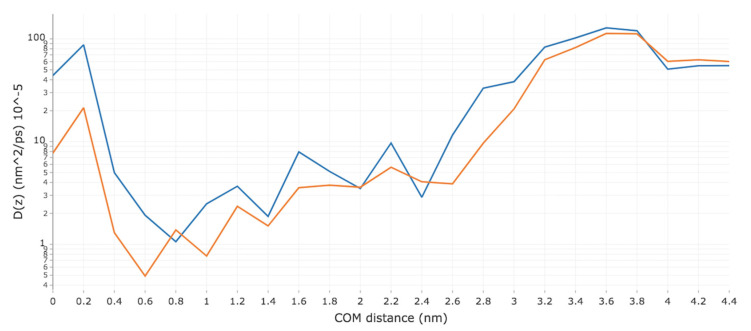
Diffusivity profile of the permeation of Me-5ALA (blue) and 5ALA (orange) through a model of the lipid bilayer of the SC.

The diffusivity profiles of both Me-5ALA and 5ALA are shown in Figure 3. As Me-5ALA approaches the bilayer surface (~2.8 nm), the diffusivity decreases significantly until it reaches the hydrophilic lipid headgroups at a distance of 2.4 nm from the COM of the bilayer. From this point onwards, the diffusivity fluctuates as Me-5ALA permeates through the high-density hydrophobic region. When Me-5ALA reaches a COM distance of 0.8 nm its diffusivity has decreased by two orders of magnitude compared to that in bulk water, reaching its minimum value. This is followed by a steep recovery as the diffusivity regains two orders of magnitude at a distance of 0.2 nm from the COM, before decreasing slightly as it reaches the middle of the bilayer. The diffusivity profile of 5ALA follows a rather similar trend. As 5ALA approaches the bilayer surface the diffusivity decreases significantly until it reaches the hydrophilic lipid headgroups at a distance of 2.6 nm from the COM of the bilayer. From this distance inwards, the diffusivity profile fluctuates (although not as much as Me-5ALA) as 5ALA permeates through the high-density hydrophobic region. When 5ALA reaches a COM distance of 0.6 nm its diffusivity has decreased by almost three orders of magnitude compared to that in bulk water. This is followed by a steep increase in diffusivity (although it does not reach the same level as Me-5ALA does) as 5ALA approaches a distance of 0.2 nm from the COM, before decreasing slightly as it reaches the middle of the bilayer.

The maximum diffusivity value and area of least resistance for both Me-5ALA and 5ALA are predicted to be in the region corresponding to bulk water (COM distance > 4.0 nm), as expected, where the diffusivity of Me-5ALA and 5ALA are very similar given the relatively minor differences in molecular weight. These molecular characteristics seem to have little to no effect on the diffusivity as the profiles of both Me-5ALA and 5ALA follow very similar trends and stay within the same order of magnitude throughout the majority of the translocation. The diffusivity of both molecules is indeed highest in the bulk water region, and second highest in the interdigitating region corresponding to areas of low density. This suggests that the diffusivity of the permeant is influenced primarily by the density of the environment, as opposed to its own individual molecular characteristics. These observations agree qualitatively with the findings reported with a phospholipid bilayer, where the diffusivity was highest in the bulk water region, decreased as the permeant travelled through the high-density lipids region, and significantly increased in the low-density interdigitating region [15]. The diffusivity profiles of Me-5ALA and 5ALA were also observed to be very similar to one another. 5ALA is a relatively hydrophilic molecule, whereas Me-5ALA is relatively more hydrophobic. Based on the diffusivity profiles shown in Figure 3, although the overall trends are the same and despite Me-5ALA having a slightly larger molecular weight, the increased hydrophobicity of Me-5ALA appears to allow it to diffuse through the lipid bilayer with consistently less resistance compared to its more hydrophilic parent compound.

### 2.3. Transdermal Permeability

The transdermal permeability of Me-5ALA across the SC is predicted to be 2.62 × 10^−11^ nm/ps and the transdermal permeability predicted for 5ALA is 6.58 × 10^−12^ nm/ps. This means that Me-5ALA is predicted to have a higher permeability than 5ALA by one order of magnitude, as expected.

Experimentally measured and computationally derived transdermal permeability values available in the literature are shown in Table 1. The transdermal permeability values of both 5ALA and Me-5ALA across real skin samples were all reported to be in the order of 10^−13^ nm/ps, despite the different sample types and experimental method used [17,22,24]. The value measured for Me-5ALA across piglet skin, which has a looser and thinner structure compared to human skin, suggest that the transdermal permeability of Me-5ALA across human skin would be significantly slower than 10^−13^ nm/ps [24]. These measurements, however, are not in agreement with the experimental values retrieved using actual human skin, which highlights the inconsistency in measured permeability coefficients across different skin samples and the potential value of computational predictions to provide more consistent estimates.

The transdermal permeability values of both 5ALA and Me-5ALA across a pure phospholipid bilayer had been reported previously to be in the order of 10^−4^ nm/ps [15,25]. This dramatic difference with respect to experimental values is attributed to the different properties that characterise different types of membranes. Unlike the dense and ordered gel state of the SC, the DPPC bilayer has low density and order resembling a fluid state [15]. This large discrepancy highlights that pure phospholipid bilayers with such properties are not appropriate or reliable models to use when predicting the permeation of molecules across the human skin because permeation across the skin occurs at much slower rates than across phospholipid bilayers, which can be seen from the measurements obtained using real skin as well as the predictions reported in this study using a more realistic model of the lipid bilayers of the SC.

### 2.4. Hydrogen-Bonding Analysis

**Figure 4:**
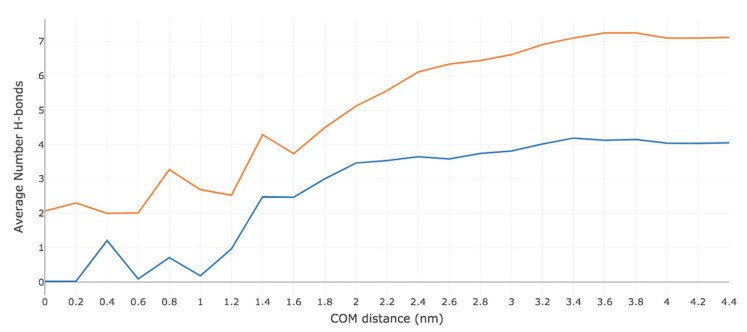
Total average number of hydrogen bonds formed by Me-5ALA and 5ALA during their permeation across a model of the lipid bilayer of the SC. The blue line represents the sum of the average number of hydrogen bonds of Me-5ALA to CER, CHOL, FFA, water and to itself. The orange line represents the sum of the average number of hydrogen bonds of 5ALA to CER, CHOL, FFA, water and to itself.

The average number of intermolecular and intramolecular H-bonds were computed at each US window for both Me-5ALA and 5ALA. Figure 4 displays the total average number of H-bonds observed between the permeant and all five components of the simulation system (i.e., CER, CHOL, FFA, water, and Me-5ALA or 5ALA). Overall, Me-5ALA participates in fewer H-bonds than 5ALA, consistent with the fact that it has a lower H-bonding capacity. A maximum of four H-bonds are observed in the bulk water region, which are all lost by the time Me-5ALA reaches a COM distance of 1.0 nm in the high-density hydrophilic lipid tail region. From this point it forms weak H-bonding interactions that are not maintained as it reaches the centre of the bilayer. On the other hand, 5ALA participates in seven H-bonding interactions in the bulk water region and loses three of these H-bonds when it enters the high-density hydrophobic tail region and maintains a minimum of two H-bonds as it reaches the centre of the bilayer.

**Figure 5:**
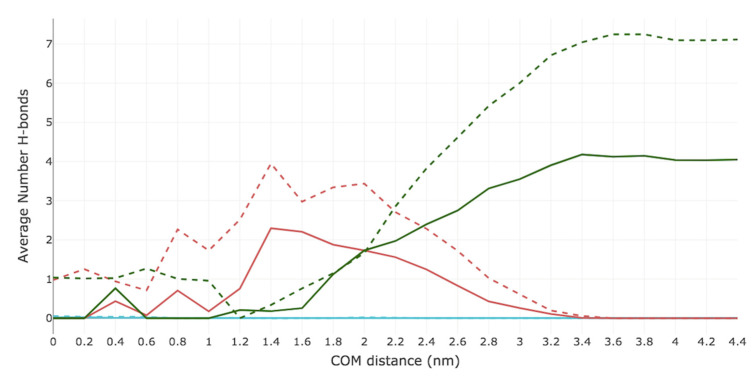
Average number of hydrogen bonds formed by Me-5ALA and 5ALA to each system component during the permeation across a model of the lipid bilayer of the SC. The solid pink line represents the average number of hydrogen bonds of Me-5ALA to lipids. The solid green line represents the average number of hydrogen bonds of Me-5ALA to water. The solid blue line represents the average number of hydrogen bonds of Me-5ALA to itself. The dotted pink line represents the average number of hydrogen bonds of 5ALA to lipids. The dotted green line represents the average number of hydrogen bonds of 5ALA to water. The dotted blue line represents the average number of hydrogen bonds of 5ALA to itself.

Figure 5 groups together the ternary lipid mixture and displays the average number of H-bonds observed between the permeant and the lipid bilayer (i.e., the combined sum of CER, CHOL, FFA), water, and itself (intramolecular). Neither of the drug molecules form intramolecular H-bonds. Me-5ALA loses all four of its H-bonds with water by the time it reaches a COM distance of 1.6 nm, and weakly interacts with a water molecule at a COM distance of 0.4 nm, which is immediately lost as it continues towards the bilayer centre. As Me-5ALA loses H-bonds with water, it forms H-bonds with the bilayer lipids, exhibiting a maximum of two H-bonds at a COM distance of 1.4 nm, which corresponds to the high-density lipid tail region, which are readily lost as Me-5ALA approaches the low-density lipid tail region. By contrast, 5ALA experiences a dramatic loss of all of its H-bonds with water, although it is predicted to regain and maintain a minimum of one H-bond with water throughout the entire translocation across the bilayer. At the same time, as 5ALA approaches the bilayer surface, the H-bonds lost with water are replaced with H-bonds with the hydrophilic lipid headgroups to prevent its polar H-bonding groups from being unsatisfied, which would be energetically unfavourable.

**Figure 6:**
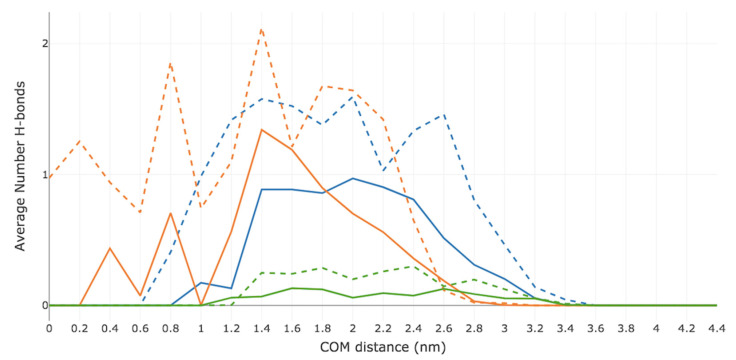
Average number of hydrogen bonds formed by Me-5ALA and 5ALA to each lipid component during the permeation across a model of the lipid bilayer of the SC. The solid blue line represents the average number of hydrogen bonds of Me-5ALA to CER. The solid orange line represents the average number of hydrogen bonds of Me-5ALA to CHOL. The solid green line represents the average number of hydrogen bonds of Me-5ALA to FFA. The dotted blue line represents the average number of hydrogen bonds of 5ALA to CER. The dotted orange line represents the average number of hydrogen bonds of 5ALA to CHOL. The dotted green line represents the average number of hydrogen bonds of 5ALA to FFA.

Figure 6 displays the average number of H-bonds observed between the permeant and each individual lipid component (i.e., CER, CHOL and FFA). Similar trends are seen in the case of both Me-5ALA and 5ALA. As the drug molecules approach the bilayer surface (at a COM of 2.8 nm) they each interact with CER, though 5ALA appears to have more H-bonding affinity for the bilayer surface. As the drug molecules traverse across the high-density lipid tail region, they both form a H-bond interaction with CHOL whilst maintaining a H-bond with CER. As Me-5ALA approaches the low-density region, it loses its H-bond with CER at a COM distance of 0.8 nm. Once it has reached the centre of the bilayer it no longer H-bonds with any of the lipid components. By contrast, 5ALA, whilst it also loses its H-bonding interaction with CER as it approaches the low-density region at a COM distance of 0.6 nm, it maintains a H-bond with CHOL throughout the entire translocation path.

## 3. Discussion

The combined prediction of free energy and diffusivity profiles, along with a detailed H-bonding analysis of the interactions of Me-5ALA and 5ALA as these drugs permeate across the model bilayer of the SC, allow the characterisation of their mechanism of permeation. First, the presence of a minimum in the free energy of interaction profiles for 5ALA and Me-5ALA reveal that both molecules have a relatively strong interaction with the hydrophilic lipid headgroups. The subsequent free energy barrier in the high-density lipid tail region for both drug molecules is not too high compared to the free energy in the bulk water region, allowing both 5ALA and Me-5ALA to continue to permeate through the lipid bilayer, where ultimately Me-5ALA has more favourable interactions within the hydrophobic core of the bilayer. Since 5ALA is predicted to maintain a H-bond with the lipid components (i.e., cholesterol) as it permeates across the entire hydrophobic lipid tail region, this explains why the free energy profile reveals that the interactions between 5ALA and the bilayer are unfavourable across the entire hydrophobic region. The H-bonding interaction between 5ALA and at least one water molecule in the high-density hydrophobic region (at a COM distance of 1.0 nm) is also reflected in the free energy profile, which exhibits the higher free energy barrier, reflective of a permeation mechanism in which 5ALA drags at least one water molecule into the hydrophobic core of the bilayer, which is ultimately an unfavourable process that is not exhibited by Me-5ALA. This distinct behaviour arises from the differences in structure and interaction properties of these drug molecules. The added methyl group in Me-5ALA not only increases the molecular weight and hydrophobicity, but also results in the loss of a H-bond donor and a reduced polar surface area of 69.4 Å^2^. By contrast, the polar surface area of 5ALA is 80.4 Å^2^, which is related to its stronger affinity for the polar hydrophilic lipid head region compared to Me-5ALA, and also the notably higher free energy barrier experienced throughout the non-polar hydrophobic tail region.

The diffusivity profiles of Me-5ALA and 5ALA show a clear decrease of one order of magnitude as the permeant comes into contact with the bilayer surface (at a COM of ~2.8 nm), a continual decrease as the permeant travels through the high-density hydrophobic region, and a recovery of two (5ALA) to three (Me-5ALA) orders of magnitude as the permeant diffuses into the interdigitating low-density region. Both drug molecules experience the least resistance against permeation and thus have greater diffusivity values in the bulk water region, which is the area with the lowest density in the simulation system, followed by the interdigitating region, which has the second lowest density, where 5ALA experiences more resistance than Me-5ALA. The additional H-bonds that 5ALA is predicted to make within the lipid bilayer explain why 5ALA has a consistently slower diffusion rate than Me-5ALA, experiencing more resistance against diffusion in the hydrophobic regions, particularly in the interdigitating hydrophobic core. Overall, this suggests that the mechanism that is responsible for Me-5ALA being a better permeant than 5ALA is due to a reduction in its ability to form hydrophilic (H-bonding) interactions and an increase in its hydrophobicity, making its permeation into the low-density non-polar region of the bilayer more favourable.

It is important to note that the transdermal permeability predicted for 5ALA is one order of magnitude faster than experimental values and is two orders of magnitude faster for Me-5ALA. This is likely due to the highly complex lipid composition present in real skin, whereas these MD simulations use a representative molecular model of the lipid layers of the SC. In real SC, molecules must also travel sideways and find gaps between the individual corneocytes through a process called lateral diffusion. This process occurs at a different rate to permeation and is likely to be a contributing factor to the difference in transdermal permeabilities, because the values predicted in this work are one-dimensional (across lipid bilayers), whereas experimental values are two-dimensional and, consequently, lower in magnitude. As Me-5ALA is a more lipophilic molecule than 5ALA, its permeation through the lipid matrix of the SC is expected to be faster, which has not yet been shown through experimental methods across the same skin sample for both drug molecules. The permeability predicted for Me-5ALA is one order of magnitude higher than for 5ALA, as expected on the basis of the differences in chemical structure, confirming that MD simulation is a useful tool that can be used to accurately predict differences in permeability between different molecules, enabling better comparisons with other compounds. This computational approach also offers an effective way of elucidating the molecular mechanism of permeation of drug molecules, such that it can provide a pathway for the rational design of other drug molecules that can permeate the SC more effectively.

## 4. Materials and Methods

### 4.1. Simulation System

**Figure 7:**
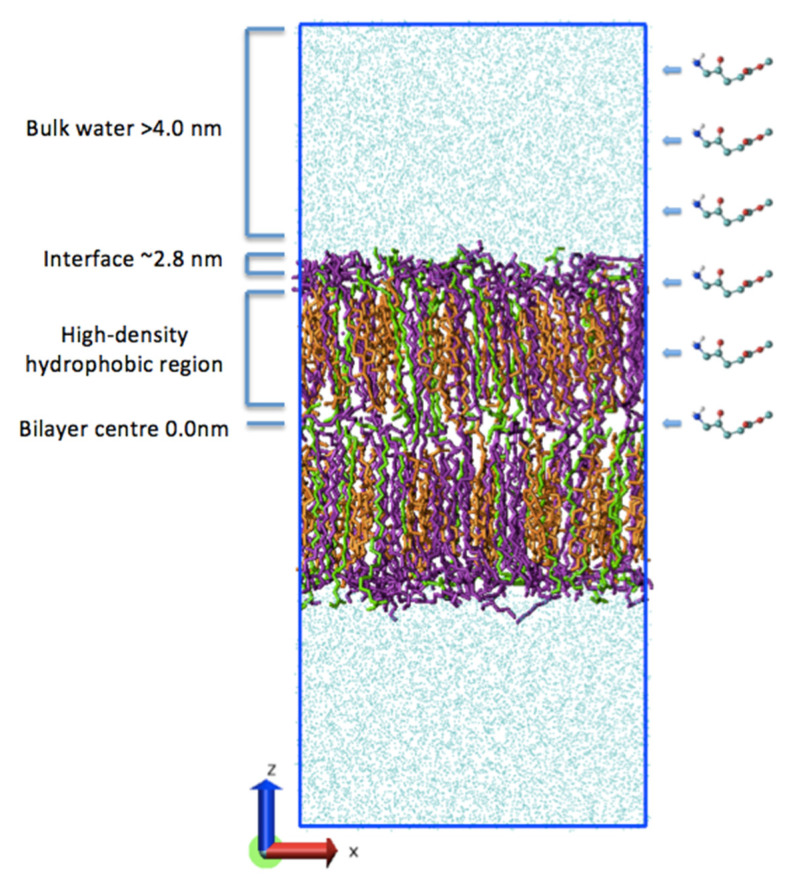
Molecular visualisation of the equilibrated lipid bilayer of the SC containing a 2:2:1 molar ratio of ceramide (purple), cholesterol (orange) and free fatty acids (green) solvated in water (blue). The permeant Me-5ALA is shown on the right-hand side at various COM distances from the bilayer centre along the z-dimension. Four key regions of the simulation system are labelled on the left-hand side.

The simulation system consisted of a ternary lipid bilayer containing ceramide (CER NS, 24:0 with sphingosine backbone C18), cholesterol (CHOL) and free fatty acid (FFA) C24 (lignoceric or tetracosanoic acid) in a 2:2:1 molar ratio, which is commonly used as a molecular model of the complex lipid composition of the SC [20]. The system comprised a total of 160 lipids, with standard molecular topologies and initial molecular geometries [37,38]. The simulation system was built using Packmol [39] and was solvated with 6000 water molecules. This fully hydrated system retains the gel-like phase of the lipid bilayers of the SC and allows the simulation of permeation here described. The lipids were arranged into two symmetrical leaflets with distinct hydrophobic and hydrophilic regions, as shown in Figure 7. The *z*-axis (longest dimension) represents the normal to the bilayer plane. The COM of the bilayer is at a position of 0.0 nm along the *z*-axis and represents the hydrophobic interdigitating region where the lipid tails of both leaflets meet. This and surrounding regions have lower density and order. Further along both sides of the COM lies the dense and ordered hydrophobic lipid tail region. The hydrophilic lipid headgroup region is lower in density towards the bilayer surface, with the bilayer–water interface located at ~2.8 nm from the COM.

The GROMOS 54A7 force field [40,41] was used to represent the intra- and inter-molecular potential energy of the lipids and the SPC model [42] was used for water. In the case of 5ALA and Me-5ALA, molecular topologies and potential energy parameters were obtained for their neutral forms from the Automated Topology Builder (ATB) [43]. The system containing the lipid bilayer in water was initially subjected to potential energy minimisation using steepest descents, before equilibration and production simulations using rectangular periodic boundary conditions (with the longer dimension corresponding to the vector normal to the bilayer plane along the *z*-axis) and a time step of 2 fs. All bond lengths were constrained using the LINCS algorithm [44]. A single-range cut-off scheme was used with a cut-off value of 1.4 nm to calculate all non-bonded interactions at every time step. For consistency with the interaction scheme used to develop the GROMOS 54A7 force field, a reaction field with a relative dielectric constant of 62 was applied to calculate electrostatic interactions beyond the cut-off. The Nosé-Hoover thermostat [45] and the semi-isotropic Parrinello-Rahman barostat [46] were used to maintain the simulation cell at physiological skin conditions of 305 K (32 °C) and 1 atmospheric pressure. The system was equilibrated for approximately 1000 ns after the area per lipid (APL) had remained stable for at least 300 ns. All simulations were conducted using an in-house version of GROMACS 4.6.7 [46] that implemented the enhanced sampling approach described further below.

### 4.2. Insertion of 5ALA and Me-5ALA in the Simulation System

The parent drug 5ALA and its prodrug Me-5ALA were each inserted in separate simulations into the bulk water region of the simulation cell and any water molecules in close vicinity (within 0.3 nm) to the permeant were removed to avoid likely steric clashes. For example, the final system with a single Me-5ALA molecule contained 5979 water molecules.

### 4.3. Pull Simulation

A pull simulation was conducted to obtain a series of initial configurations of each of the permeant molecules at different depths of the bilayer. These simulations were conducted for 10 ns as the permeant molecule was pulled along the z-direction of the simulation cell with a harmonic force constant of 125 kJ mol^−1^ nm^−2^ from the bulk water region (COM distance >4.0 nm) towards the lipid bilayer to reach different COM distances. This process created multiple starting configurations for subsequent enhanced sampling simulations.

### 4.4. Umbrella Sampling and Selective Replica Exchange with Solute Tempering

The actual permeation of molecules across the SC is very slow compared to accessible MD simulation times, requiring the use of enhanced sampling methods to properly represent the relevant molecular events (translations, rotations and molecular conformations) involved in this process. The commonly used umbrella sampling (US) method was used for this purpose. This method uses a series of independent simulations that are referred to as windows, each representing the permeant at a different insertion depth through the bilayer (the so-called reaction coordinate). A total of 23 US windows (simulations) were implemented, corresponding to a series of COM distances ranging from 0.0 nm (centre of the lipid bilayer) to 4.4 nm (bulk water region). These windows were spaced 0.2 nm apart to allow for slight overlap of the positions of the permeant along the reaction coordinate (*z*-axis). The series of configurations obtained from the above-described pull simulations represent the entire translocation pathway of the permeant and were used as the starting configurations for the US window simulations. A harmonic force constant of 500 kJ mol^−1^ nm^−2^ was applied to restrain the position of the COM of the permeant in each US window.

To further enhance the sampling of molecular events at each COM distance, a modification of the well-known replica exchange with solute tempering (REST and REST2) methods [47,48] was implemented to selectively temper the non-bonded interactions (van der Waals and electrostatic interactions) between the solute (i.e., the permeant) and the lipid bilayer (but not other simulation components) using different interaction scaling factors, a method henceforth referred to as REST3. This approach allows separation of the scaling of the permeant-solvent (unperturbed) and permeant-bilayer (tempered) interactions, further allowing the interactions between and within the solvent and the bilayer lipid molecules to remain unperturbed, as well as within the permeant molecule. This approach is ideally suited for the simulation of the interaction and permeation of small molecules across lipid bilayers because it allows interactions that maintain the integrity of lipid bilayers (i.e., lipid–lipid and lipid–solvent interactions) to remain unchanged. The REST approach is equivalent to increasing the temperature (tempering) of the relevant species, such that in the case of REST3 the permeant behaves as though it were at higher temperatures, allowing it to escape long-lived interactions with the lipid bilayer and sample other relevant configurations more effectively.

The REST3 method is applied across multiple replicas in each US window of the system, each replica with varying scaling (tempering) factors, referred to as the replica ladder. The replica with a scaling factor of 1.0 is referred to as the ground replica as it has no downscaling and thus, all non-bonded interactions remain at 100% of their strength. The gradual decrease in scaling factor results in stronger tempering and, hence, a reduction in the strength of non-bonded interactions. REST3 simulations were done with an exchange attempt frequency set to 0.1 ps and replica exchange acceptance percentages between 20 and 30%. Table A1 in Appendix A summarises the three replica schemes that were used, along with the number of replicas of the system that were run in parallel, and the scaling factors used. Each of the US windows with a COM distance of 0.0 nm to 3.8 nm had one of the three replica schemes applied to make sure that there was sufficient sampling of the molecular events at each COM distance along the reaction coordinate. There were a total of 176 replicas of the system across 20 US+REST3 simulation windows. The high-density lipid tail region required the most tempering because it is the region where the permeant is most unable to freely translate, rotate and change conformation. The US windows corresponding to the bulk water region (COM distance ≥4.0 nm) did not have the REST3 method applied as the permeant does not interact with the bilayer in this region and hence additional enhanced sampling was not necessary. All replica simulations in each window were run for 250 ns each. The total simulation time of all 23 windows was 44,750 ns (44.75 μs) for each permeant.

### 4.5. Convergence of the Free Energy of Permeation

Interaction of the permeant with the lipid bilayer at different insertion depths along the reaction coordinate results in changes in the free energy of the system. These position-dependent changes in free energy can be combined to give a free energy profile, often referred to as the potential of mean force (PMF), which measures the relative changes in free energy across the entire translocation pathway of the permeant. The PMF was retrieved using the weighted histogram analysis method (WHAM) in GROMACS through the g_wham module [49]. The free energy was set to zero in the bulk water region (COM distance ≥4.0). The standard deviation of the PMF calculation was obtained using bootstrapping with 200 iterations.

The convergence time of the simulation was determined by plotting the computed PMF of the simulation trajectory in 50 ns blocks at progressively later start times in increments of 10 ns (i.e., 0 to 50 ns, 10 to 60 ns simulation, 20 to 70 ns simulation, for up to 200 to 250 ns). The convergence time reflects the time taken for the simulation to produce a stable PMF and is therefore disregarded when reporting the predicted PMF of each permeant system. The long convergence time required (i.e., 210 ns) is a consequence of the time needed for molecular configurations in high-tempered replicas to reach a steady-state of exchange with the ground replica across all windows. The final equilibrium PMF was determined by analysing the final 40 ns of the simulation of each permeant.

### 4.6. Estimation of Diffusivity Profiles

The diffusivity of the permeant was retrieved using Hummer’s derivation, which relates the position autocorrelation function to the friction (and, thus, its diffusivity) experienced by a permeant under a US harmonic restraint [50]. The diffusion coefficient for each COM distance (i.e., each US window) was calculated as the ratio of the variance of the time series of COM positions in the z-direction and the integrated autocorrelation time (IACT) [51], with the latter retrieved using the GROMACS g_wham module.

### 4.7. Permeability Coefficient and Transdermal Permeability

The permeability coefficient was calculated from the free energy and diffusivity values using the following equation:

(1) $$\frac{1}{P}=\frac{\int_{z_{1}}^{z_{2}}e^{\frac{\Delta G\left(\xi \right)}{RT}}d\xi }{D\left(\xi \right)}.$$

Here *P* represents the permeation coefficient, *ξ* represents the position of the permeant relative to the bilayer, *ΔG* represents the relative free energy (from the PMF), *D* represents the position dependent diffusion coefficient and *T* represents the temperature. The transdermal permeability was then calculated using the following equation:

(2) $$\frac{1}{P}=\frac{1}{P_{1}}+\frac{1}{P_{2}}+\ldots +\frac{1}{P_{200}}.$$

This equation was used to accommodate for the multi-layered structure of the SC, assuming that it has a typical number of ten corneocyte layers, each surrounded by ten lipid bilayers, therefore totalling 100 lipid bilayers in the SC, i.e., 200 lipid layers (leaflets).

### 4.8. Hydrogen-Bonding Analysis

Analysis of hydrogen bonds (H-bonds) was used to describe the molecular interactions between the permeant and the bilayer and the aqueous solvent. The GROMACS g_hbond module was used to compute simulation time averages with a H-bond donor–acceptor distance cut-off of 0.35 nm and a hydrogen–donor–acceptor angle of 37° between all of the potential H-bond donors and acceptors.

## 5. Conclusions

MD simulations predicted that Me-5ALA has a higher permeability than its parent compound 5ALA across a model lipid bilayer of the SC. Me-5ALA has an additional methyl group that removes one H-bond donor compared to 5ALA and reduces its polar surface area by 11 Å^2^. These differences in chemical structure reduce the H-bonding capability of Me-5ALA and increase its hydrophobicity, allowing it to better permeate across the SC. The main differences observed between Me-5ALA and 5ALA manifest in the hydrophobic core of the lipid bilayer, corresponding to the low-density lipid tail region. This region exhibits a favourable free energy for the permeation of Me-5ALA due to the lack of hindering H-bonding interactions that 5ALA exhibits with the lipids and water, ultimately allowing Me-5ALA to diffuse at a faster rate and with less resistance across the bilayer.

This comparative study of the prodrugs Me-5ALA and 5ALA is a model system for the characterisation of the influence of surface polarity of drug molecules and, in particular, the potential use of a polar-to-non-polar ratio as a molecular descriptor for the comparative analysis of the permeation of drugs across lipid bilayers. The use of MD simulations to characterise the influence of the increased hydrophobicity of the prodrug Me-5ALA on its permeation across the skin demonstrate the usefulness of this approach to elucidate the molecular mechanism of permeation of drugs across lipid bilayers for the rational design of drugs with enhanced transdermal permeation.

## Figures and Tables

**Table 1:** Transdermal permeability values for Me-5ALA and 5ALA.

Molecule	Sample Type	Method	P (nm/ps)
5ALA	Human thigh skin	Horizontal-type diffusion cell	2.25 × 10^−13^
5ALA	Human abdomen skin	Franz-type diffusion cell	2.36 × 10^−13^
Me-5ALA	Piglet skin	Franz-type diffusion cell	4.17 × 10^−13^ (Solution) 7.50 × 10^−13^ (Gel)
5ALA	DPPC bilayer	MD simulation	1.89 × 10^−4^
Me-5ALA	DPPC bilayer	MD simulation	5.28 × 10^−4^
5ALA	SC lipid bilayer	MD simulation (this work)	6.58 × 10^−12^
Me-5ALA	SC lipid bilayer	MD simulation (this work)	2.62 × 10^−11^

## Data Availability

All the data pertaining to this study (input and trajectory output files) are publicly available in Zenodo at https://doi.org/10.5281/zenodo.7316440 (published on 13 November 2022) (for 5-ALA) and https://doi.org/10.5281/zenodo.7316897 (published on 13 November 2022) (for Me-5ALA).

## Appendix A

**Table A1:** Number of replicas, scaling factors and simulation time used for each US window.

US Window (COM Distance/nm)	Replica Scheme		Time (ns)
	No. of Replicas	Scaling Factors	
0.0	5	1.00, 0.88, 0.75, 0.63, 0.50	1250
0.2	5	1.00, 0.88, 0.75, 0.63, 0.50	1250
0.4	5	1.00, 0.88, 0.75, 0.63, 0.50	1250
0.6	5	1.00, 0.88, 0.75, 0.63, 0.50	1250
0.8	11	1.00, 0.91, 0.82, 0.73, 0.64, 0.55, 0.46, 0.37, 0.28, 0.19, 0.10	2750
1.0	11	1.00, 0.91, 0.82, 0.73, 0.64, 0.55, 0.46, 0.37, 0.28, 0.19, 0.10	2750
1.2	11	1.00, 0.91, 0.82, 0.73, 0.64, 0.55, 0.46, 0.37, 0.28, 0.19, 0.10	2750
1.4	11	1.00, 0.91, 0.82, 0.73, 0.64, 0.55, 0.46, 0.37, 0.28, 0.19, 0.10	2750
1.6	11	1.00, 0.91, 0.82, 0.73, 0.64, 0.55, 0.46, 0.37, 0.28, 0.19, 0.10	2750
1.8	11	1.00, 0.91, 0.82, 0.73, 0.64, 0.55, 0.46, 0.37, 0.28, 0.19, 0.10	2750
2.0	10	1.00, 0.90, 0.80, 0.70, 0.60, 0.50, 0.40, 0.30, 0.20, 0.10	2500
2.2	10	1.00, 0.90, 0.80, 0.70, 0.60, 0.50, 0.40, 0.30, 0.20, 0.10	2500
2.4	10	1.00, 0.90, 0.80, 0.70, 0.60, 0.50, 0.40, 0.30, 0.20, 0.10	2500
2.6	10	1.00, 0.90, 0.80, 0.70, 0.60, 0.50, 0.40, 0.30, 0.20, 0.10	2500
2.8	10	1.00, 0.90, 0.80, 0.70, 0.60, 0.50, 0.40, 0.30, 0.20, 0.10	2500
3.0	10	1.00, 0.90, 0.80, 0.70, 0.60, 0.50, 0.40, 0.30, 0.20, 0.10	2500
3.2	10	1.00, 0.90, 0.80, 0.70, 0.60, 0.50, 0.40, 0.30, 0.20, 0.10	2500
3.4	10	1.00, 0.90, 0.80, 0.70, 0.60, 0.50, 0.40, 0.30, 0.20, 0.10	2500
3.6	10	1.00, 0.90, 0.80, 0.70, 0.60, 0.50, 0.40, 0.30, 0.20, 0.10	2500
3.8	10	1.00, 0.90, 0.80, 0.70, 0.60, 0.50, 0.40, 0.30, 0.20, 0.10	2500
4.0	0	NA	250
4.2	0	NA	250
4.4	0	NA	250
